# Nanoparticulate iron(III) oxo-hydroxide delivers safe iron that is well absorbed and utilised in humans

**DOI:** 10.1016/j.nano.2014.06.012

**Published:** 2014-11

**Authors:** Dora I.A. Pereira, Sylvaine F.A. Bruggraber, Nuno Faria, Lynsey K. Poots, Mani A. Tagmount, Mohamad F. Aslam, David M. Frazer, Chris D. Vulpe, Gregory J. Anderson, Jonathan J. Powell

**Affiliations:** a MRC Human Nutrition Research, Elsie Widdowson Laboratory, Cambridge, United Kingdom; b Department of Nutritional Science and Toxicology, University of California, Berkeley, CA, USA; c Iron Metabolism Laboratory, QIMR Berghofer Medical Research Institute, PO Royal Brisbane Hospital, Brisbane, Australia

**Keywords:** Ligand-modified Fe(III) poly oxo-hydroxide, Iron supplementation, Bioavailability, Microbiota, Redox damage

## Abstract

Iron deficiency is the most common nutritional disorder worldwide with substantial impact on health and economy. Current treatments predominantly rely on soluble iron which adversely affects the gastrointestinal tract. We have developed organic acid-modified Fe(III) oxo-hydroxide nanomaterials, here termed nano Fe(III), as alternative safe iron delivery agents. Nano Fe(III) absorption in humans correlated with serum iron increase (*P* < 0.0001) and direct in vitro cellular uptake (*P* = 0.001), but not with gastric solubility. The most promising preparation (iron hydroxide adipate tartrate: IHAT) showed ~80% relative bioavailability to Fe(II) sulfate in humans and, in a rodent model, IHAT was equivalent to Fe(II) sulfate at repleting haemoglobin. Furthermore, IHAT did not accumulate in the intestinal mucosa and, unlike Fe(II) sulfate, promoted a beneficial microbiota. In cellular models, IHAT was 14-fold less toxic than Fe(II) sulfate/ascorbate. Nano Fe(III) manifests minimal acute intestinal toxicity in cellular and murine models and shows efficacy at treating iron deficiency anaemia.

**From the Clinical Editor:**

This paper reports the development of novel nano-Fe(III) formulations, with the goal of achieving a magnitude less intestinal toxicity and excellent bioavailability in the treatment of iron deficiency anemia. Out of the tested preparations, iron hydroxide adipate tartrate met the above criteria, and may become an important tool in addressing this common condition.

## Graphical Abstract

Current oral treatment of iron deficiency anaemia is based upon soluble iron compounds, all of which have undesirable gastrointestinal effects. However, natural dietary iron (III) may be nano-formed ligand-modified iron hydroxide particles that are acquired endocytically and thus *not* soluble or available for adverse effects. Here, a series of five different nano Fe(III) hydroxide particles was developed: absorption in humans matched endocytic uptake by gut epithelial cells but not solubility in (stomach) acid. In murine and cellular models nano iron(III) treated iron deficiency anaemia as well as ferrous sulfate but, unlike the latter, was safe to the gut environment.

**Figure:**
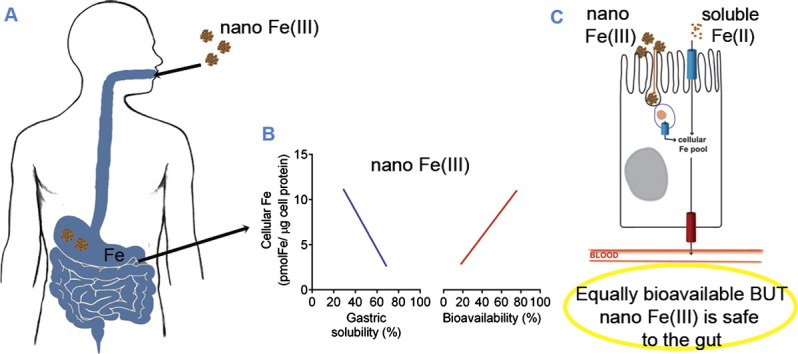

Anaemia is one of the World Health Organization's top 10 target diseases for cure and prevention.^1^ However, despite considerable global efforts with oral iron supplementation and fortification, iron deficiency remains the most common and widespread nutritional disorder in the world, affecting 4 billion people.^2^, ^3^, ^4^, ^5^, ^6^ Achieving practicable strategies for iron fortification, ensuring the bioavailability of the iron used and obviating the side effects of oral iron supplements, are some of the well-known challenges for fortification/supplementation programs. However, in recent years, more significant questions are being posed by safety data outcomes from oral iron studies. A negative impact on the commensal flora, especially suppression of *Lactobacillus*,^7^ and an enhancement of systemic infection for at-risk populations^8^, ^9^ have been convincingly demonstrated in oral iron supplementation/fortification studies in humans. Most recently, a marked enhancement of colonic carcinogenesis by soluble luminal (chelated) iron has been demonstrated in mice,^10^ including those with *Apc* mutations, the commonest gene mutation in sporadic colon cancer in humans.^11^

The physicochemical forms of iron that are commonly used for supplementation and/or fortification may contribute to significant undesirable effects. Iron that remains soluble in the intestinal lumen is likely to be bioavailable but, equally, may be available for other processes including uptake by commensal flora and colonic epithelial cells, and facile redox-cycling in the gut lumen. Indeed, the generation of harmful free-radicals through Fenton chemistry which can cause inflammation and oxidative stress in the intestinal mucosa has been linked to available luminal iron in several studies. Carrier et al have shown that in rats with DSS-induced colitis, ferrous sulfate supplementation increased colonic and plasma lipid peroxides and overall disease activity.^12^ Increased oxidative stress following oral iron therapy has been supported in clinical studies in inflammatory bowel disease^13^ and in healthy subjects.^14^, ^15^ Fe(II) sulfate was also shown to be involved in the onset of chronic disease in a murine ileitis model.^16^ In the DSS-induced colitis mouse model, Seril and colleagues found a remarkable increase in tumour incidence (from 19 to 88%) by doubling the amount of iron in the diet using sodium Fe(III) EDTA.^10^ Furthermore, recent reports of a transient increase in undesirable non-transferrin bound iron (NTBI)^12^, ^14^, ^15^, ^16^, ^17^, ^18^ in the systemic circulation after oral supplementation with soluble iron may be a consequence of iron entering the circulation at a rate that exceeds the rate of transferrin binding. NTBI has been associated with an increased risk of infection and coronary heart disease.^19^, ^20^, ^21^, ^22^

Recently, we have developed 5-10 nm hydro-disperse particles of iron oxo-hydroxide that are modified synthetically with the addition of tartaric and adipic acids, and mimic^23^, ^24^, ^25^ the well-absorbed ferritin core.^26^, ^27^, ^28^, ^29^, ^30^, ^31^ These same ligand-modified nano iron oxo-hydroxide particles [i.e. with tartaric (T) and adipic (A) acids used as ligands at a ratio 1:1:2 (T:A:Fe)] have been shown in cellular and animal models to be taken up by intestinal epithelial cells via a mechanism independent of luminal redox activity and, therefore, independent of apical DMT1.^23^, ^25^ Uptake, probably, is by endocytosis followed by lysosomal dissolution to release bioavailable iron,^25^ although the precise pathway is incompletely elucidated. Importantly, we have shown in murine models that absorption of iron from these modified nano iron oxo-hydroxide particles is regulated by normal iron homeostasis mechanisms: i.e. through the action of hepcidin on the iron exporter ferroportin.^24^ Data in humans, however, are lacking. Hence, in this work, we have engineered five different structures with near identical physical properties (size) but divergent chemical properties (acid solubility) to determine (a) the bioavailability of these synthetic Fe(III) nanomaterials in volunteers, (b) the role of gastric acid dissolution *versus* direct cellular uptake of the nanoparticles in determining bioavailability and (c) the rates of absorption *versus* Fe(II) sulfate. Finally, we have made some initial safety measurements of the most bioavailable nano Fe(III) material and we conclude that this ‘ferritin core mimetic’ may provide a solution for safe and efficacious oral iron supplementation.

## Methods

### Iron materials

Non enteric-coated Fe(II) sulfate tablets (Actavis, Barnstaple, UK), equivalent to 60 mg Fe/tablet, were purchased from a local pharmacy. The nanoparticulate ligand-modified Fe(III) poly oxo-hydroxides, here referred to as nano Fe(III), were produced using food grade reagents following the protocol described by Powell et al^23^ Further synthetic and analytical details are supplied in the Supplementary Materials. The ligand composition and particle size of each of the five different nano Fe(III) materials investigated as well as unmodified iron oxo-hydroxide (synthetic ferrihydrite) are presented in Table 1.

**Table 1:** Characteristics of the Fe(III) oxo-hydroxide materials investigated.

Test compound	Ligands	Molar ratio of ligands: Fe	Physical form	Hydrodynamic diameter in H_2_O^i^ (nm)	
				Mean	D(v)0.1 D(v)0.9
Nano Fe(III) (*a*)	Tartaric acid (T) Adipic acid (A)	1:1:2 (T:A:Fe)	Dry powder	5.0 (0.4)	3.0 (0.7) 7.70 (0.08)
Nano Fe(III) (*b*)	Tartaric acid (T) Succinic acid (S)	1:1:2 (T:S:Fe)	Dry powder	4.9 (0.2)	2.9 (0.2) 7.6 (0.2)
Nano Fe(III) (*c*)	Tartaric acid (T) Succinic acid (S)	1:6:2 (T:S:Fe)	Dry powder	7.3 (0.5)	3.7 (1.2) 11.6 (0.5)
Nano Fe(III) (*d*)	Gluconic acid (G) Adipic acid (A)	1:1:2 (G:A:Fe)	Dry powder	2.88 (0.09)	1.76 (0.09) 4.4 (0.1)
Nano Fe(III) (*e*)^ii^	Tartaric acid (T) Adipic acid (A)	1:1:2 (T:A:Fe)	Aqueous suspension	4.80 (0.09)	2.8 (0.2) 7.39 (0.07)
Fe(III) (OH)_3_iii	N/A	N/A	Dry powder	> 1000 (agglomerated)	

N/A—not applicable.

i Nano Fe(III) (*a*)–(*d*) and Fe(III) (OH)_3_ dried materials were resuspended in H_2_O_UHP_ at [Fe] = 8 mM and nano Fe(III) (*e*) was diluted in H_2_O_UHP_ to [Fe] = 8 mM. All nano Fe(III) materials were sonicated for 5 minutes and then centrifuged at 4000 × *g* for 5 minutes and filter-sterilized through 0.2 μm filters prior to nanosizing. Micron-sized Fe(III)(OH)_3_ was sonicated and centrifuged under the same conditions but was not filtered prior to nanosizing. Data are presented as mean (SD) hydrodynamic diameter of 3 measurements, with the lower 10% [d(v)0.1 (SD)] and upper 90% [d(v)0.9 (SD)] percentile values.

ii Nano Fe(III) (e) is the same formulation as nano Fe(III) (a), but was used in the human study in a colloidal suspension (i.e. as synthesised) without drying.

iii Refers to standard unmodified synthetic Fe(III) oxo-hydroxide (i.e. synthetic 2-line ferrihydrite).

### Acid lability

The solubility of the iron materials was determined at pH 3.0 (lower end of the pH range of the postprandial gastric environment^32^) using an autotitrator. Further details are provided in the Supplementary Materials.

### Caco-2 iron uptake studies

Cellular uptake studies were carried out in Caco-2 cells as described previously,^25^ and specific full details are in the Supplementary Materials. Values for uptake are reported as total cellular Fe content, and include not only Fe that is internalised by the cell but also Fe that remains associated with the cell membrane after washing. All data were normalised to total cell protein content and corrected for control levels (i.e. levels in cells incubated with BSS not supplemented with iron). The soluble iron material used as a control in the cellular assays was Fe(III) maltolate (Supplementary Materials). Baseline ferritin levels in control cells not exposed to iron were between 2 and 10 ng/mg cell protein in line with values previously reported for non-iron supplemented Caco-2 cells.^33^, ^34^ Cellular iron levels in these same control cells were negligible (i.e. below the ICP-OES detection limit of 0.05 μM once digested and diluted for analysis—i.e. below 1.25 μM or 1 pmol/μg cell protein in undiluted cell lysate). The background iron content of BSS not supplemented with iron was 0.0010 ± 0.0009 mM and this was negligible in relation to the iron content added to the media (i.e. 0.5 mM).

### Cell viability assay

Cell viability was assessed in Caco-2 and HT-29 cells using a tetrazolium-based colorimetric assay as detailed in the Supplementary Materials. A mixture of Fe(II) sulfate and ascorbate (molar ratio 1:10) was used as a positive control in Caco-2 cells.

### Animal study

This study was carried out in strict accordance with the Australian Code of Practice for the Care and Use of Animals for Scientific Purposes. Every effort was made to minimise suffering. All animal procedures were approved by the QIMR Berghofer Medical Research Institute Animal Ethics Committee. Twenty one day old male Sprague–Dawley rats (*n* = 6) were housed individually and fed *ad libitum* an iron deficient diet (iron content 3 to 5 mg/kg wet weight)^35^ for 6 weeks prior to the start of the study (see Supplementary Table S1 for the diet composition). Following the iron depletion period, the animals were administered *ad libitum* one of the two test diets (*n* = 3 per group) for 14 days. The test diets were equivalent to the Fe deficient diet,^35^ but were supplemented with either 20 mg Fe/kg diet as Fe(II) sulfate or 20 mg Fe/kg diet as nano Fe(III). Three additional rats were fed an Fe-sufficient diet (Fe content: 50 mg Fe/kg diet as Fe(III) citrate^35^) *ad libitum* throughout the whole study and were used for comparison in the histological assessment. Further details are in the Supplementary Materials.

#### Faecal microbiota analysis

The composition of the faecal microbiota was determined using 454-pyrosequencing as described in Supplementary Materials.

## Human study

### Design and subjects

Bioavailability of the different nano Fe materials was determined in a single-dose iron absorption study in mild-moderately iron deficient, pre-menopausal female subjects (18–45 years). The study was approved by the U.K. National Research Ethics Service (06/Q0102/47) and carries ClinicalTrials.gov registration number, NCT01991600. Written individual informed consent was obtained prior to enrolment in the study. The demographic and anthropometric characteristics of study participants are presented in the Supplementary Table S2. Eligible participants were invited to two study visits (day 1 and day 14). The participants ingested, on day 1 of the study, one of the test nano iron formulations (58 ± 7 mg elemental iron equivalent), and 14 days later, one Fe(II) sulphate tablet (60 mg elemental iron equivalent), which is still the gold standard of oral iron therapy. Five ligand-modified Fe(III) oxo-hydroxide materials [nano Fe(III) (*a-e*)] (*n* = 4 per group) were tested alongside unmodified Fe(III) oxo-hydroxide [Fe(III)(OH)_3_] (*n* = 2) and a control mixture containing Fe(III) chloride, tartaric and adipic acids (*n* = 4) in the same quantities as those used for nano Fe(III) (*a*). Twenty six women completed the study. Details of the eligibility criteria, blood analysis and study visit protocols are provided in Supplementary Materials. Absorption of the nano Fe(III) materials was determined by erythrocyte incorporation of ^58^Fe^36^ as described in Supplementary Materials. Relative bioavailability values (RBV) for nano Fe(III) were determined by standard methodology, namely dividing iron absorption from nano Fe(III) by iron absorption from ferrous sulfate for each study subject. RBV are expressed in percentage.

## Results

Five different nano Fe(III) oxo-hydroxide materials were investigated as potential iron supplements: to achieve this, the native oxo-hydroxide structure of these materials (i.e. synthetic ferrihydrite) was modified by the purposeful incorporation of small organic ligands (see Table 1 as detailed in Methods). All materials were fine nanoparticulate structures in aqueous suspension, with mean hydrodynamic diameters < 10 nm, and thus they were too small to allow particle charge (i.e. zeta potential) measurements.

## Solubility at low pH

**Figure 1:**
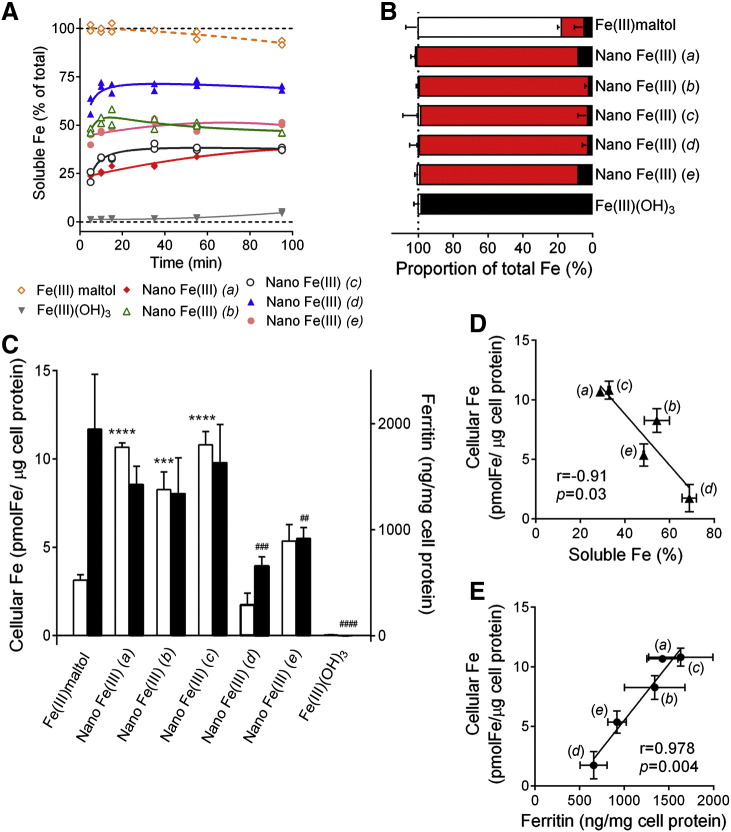
**Solubility and cellular uptake of nano Fe(III). (A)** Acid dissolution at pH 3.0 in 9 g/L NaCl. Data are for different formulations of ligand-modified Fe(III) oxo-hydroxides: nano Fe(III) *(a)*- ligands are tartaric (T) and adipic (A) acids at a ratio 1:1:2 (T:A:Fe) and the material was dried prior to re-suspension; nano Fe(III) *(b)*—ligands are tartaric (T) and succinic (S) acids at a ratio 1:1:2 (T:S:Fe) and the material was dried prior to re-suspension; nano Fe(III) *(c)*- ligands are tartaric (T) and succinic (S) acids at a ratio 1:6:2 (T:S:Fe) and the material was dried prior to re-suspension; nano Fe(III) *(d)*- ligands are gluconic (G) and adipic (A) acids at a ratio 1:1:2 (G:A:Fe) and the material was dried prior to re-suspension; nano Fe(III) *(e)*- ligands are tartaric (T) and adipic (A) acids at a ratio 1:1:2 (T:A:Fe) and the material was used as a colloidal suspension (i.e. as synthesised) without drying (more details in Table 1). Negative and positive controls are, respectively, unmodified Fe(III) oxo-hydroxide (Fe(III)(OH)_3_) and Fe(III) maltolate (Fe(III) maltol). Data are shown for the two independent replicates. Dotted black lines show 0 and 100% solubility. All data were obtained by measuring the iron concentration in the supernatant following ultrafiltration (M_r_ 3000 cut-off). **(B)** Dispersion of the different iron materials in the BSS uptake medium, used for the Caco-2 cell experiments, as assessed by the fractional percentage of microparticulate (black), nanoparticulate (red) and soluble (white) Fe for each Fe material. Values are mean ± SD of three independent replicates. **(C)** Cellular iron (open bars) and ferritin (closed bars) levels in Caco-2 cells 23 hours following a one hour exposure to 0.5 mM Fe as unmodified Fe(III) oxo-hydroxide (Fe(III)(OH)_3_), ligand-modified Fe(III) oxo-hydroxides (nano Fe(III) (*a-e*)), or soluble Fe(III) maltolate (Fe(III)maltol). Results are mean ± SD of three independent experiments (each condition tested in triplicate wells within each experiment). Statistical comparisons in relation to the soluble control, Fe(III) maltol: ***, *P* = 0.0008; ****, *P* < 0.0001 for cellular iron; ^##^, *P* = 0.003; ^###^, *P* = 0.0002 and ^####^, *P* < 0.0001 for ferritin. **(D)** Pearson's correlation between the solubility of nano Fe(III) at pH3.0 after 15 minutes and cellular iron levels of Caco-2 cells following exposure to nano Fe(III). **(E)** Pearson's correlation between cellular ferritin levels and cellular iron levels in Caco-2 cell monolayers following exposure to nano Fe(III). For panels **(D)** and **(E)**, values are mean ± SD, in both the X and Y directions. Where not apparent, the error bars are smaller than the symbol size. Data points are labelled with the nano Fe(III) preparation codes (*a–e*).

It is commonly considered that an effective iron supplement must be soluble under the conditions found in the gastrointestinal tract and, typically, this is achieved in the stomach. Using a low pH to simulate gastric dissolution, the differing nano Fe(III) materials showed ~ 3-fold variation in absolute solubility at 90 minutes. Initial dissolution was always very rapid (within the minimum of 5 minutes required to set up the assay) and was then very slow or plateaued across the remaining 90 minutes (Figure 1, *A*). Acid solubility of the positive control material [soluble Fe(III) maltolate] and the negative control [unmodified Fe(III) oxo-hydroxide] (i.e. synthetic ferrihydrite) were, as expected, 100% and minimally soluble respectively (Figure 1, *A*).

## Cellular uptake and utilisation and relationship to acid solubility

All nano Fe(III) materials were > 95% dispersed when added to the cell culture medium (Figure 1, *B*). As expected, Fe(III) maltolate was almost fully soluble and the unmodified Fe(III) oxo-hydroxide was insoluble (Figure 1, *B*). Surprisingly, acid solubility (Figure 1, *A*) of the nano Fe(III) materials correlated *inversely* with their uptake by Caco-2 cells (Figure 1, *C* and *D*; *r* = − 0.91, *P* = 0.03). However, cellular utilisation (i.e. ferritin formation by the cells) correlated very closely with iron uptake by Caco-2 cells (Figure 1, *E*; *r* = 0.978, *P* = 0.004).

## Oral bioavailability of the nano Fe (III) supplements in humans and relationship to *in vitro* characteristics

**Figure 2:**
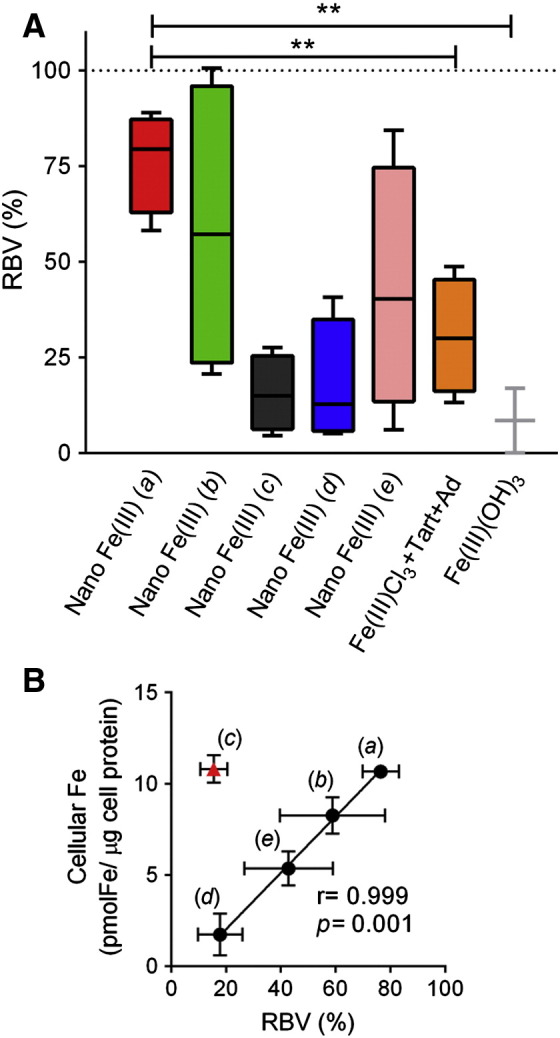
**Absorption of iron from nano Fe(III) in iron-deficient women. (A)** Relative bioavailability values (RBV) in relation to Fe(II) sulfate (100%). Percentage RBV for the nano Fe(III) preparations was calculated from the incorporation of labelled ^58^Fe into red blood cells, as measured by ICP-MS 14 days after ingestion of a single-dose of labelled compound (60 mg elemental Fe). Absorption from Fe(II) sulfate was estimated from the serum Fe curve with validated algorithms.^39^, ^66^ Nano Fe(III) (*a*)—ligands are tartaric (T) and adipic (A) acids at a ratio 1:1:2 (T:A:Fe) and the material was dried prior to re-suspension; nano Fe(III) (*b*)—ligands are tartaric (T) and succinic (S) acids at a ratio 1:1:2 (T:S:Fe) and the material was dried prior to re-suspension; nano Fe(III) (*c*)—ligands are tartaric (T) and succinic (S) acids at a ratio 1:6:2 (T:S:Fe) and the material was dried prior to re-suspension; nano Fe(III) (*d*)— ligands are gluconic (G) and adipic (A) acids at a ratio 1:1:2 (G:A:Fe) and the material was dried prior to re-suspension; nano Fe(III) (*e*)—ligands are tartaric (T) and adipic (A) acids at a ratio 1:1:2 (T:A:Fe) and the material was used as a colloidal suspension (i.e. as synthesised) without drying (more details in Table 1). Controls are unmodified Fe(III) oxo-hydroxide (Fe(III)(OH)_3_) and an ‘unformulated’ mixture of Fe(III) chloride, tartaric acid and adipic acid in the same ratios as those used in nano Fe(III) (*a*). Box and whisker plots show median, minimum and maximum for *n* = 2 (Fe(III) (OH)_3_) or *n* = 4 (all other iron materials). **, *P* = 0.004. **(B)** Pearson's correlation between cellular iron levels in Caco-2 cells exposed to nano Fe(III) (preparations *a-e*) and relative bioavailability values (%RBV) of the same materials, as shown in Figures 1, *C* and 2, *A*. Nano Fe(III) (*c*) (shown in red triangle) was excluded from the correlation parameters presented in the panel (see Results and Discussion). Values are shown as mean ± SD in both the X and Y directions. Data points are labelled with the nano Fe(III) preparation codes (*a-e*).

Using the gold standard measurement of isotopic incorporation into haemoglobin we confirmed that, as previously reported in human and rodent feeding studies,^23^, ^37^, ^38^ Fe(III) oxo-hydroxide that had not been ligand-modified (i.e. unmodified synthetic ferrihydrite) was poorly absorbed/utilised in iron deficient subjects [*P* < 0.0001 *versus* Fe(II) sulfate]. Its bioavailability was 5-fold lower than the average of the ligand-modified nano Fe(III) materials (Figure 2, *A*).

The utilisation of iron following oral dosing of the nano Fe(III) materials in iron deficient subjects (Figure 2, *A*) followed the predicted utilisation from cellular experiments, with the exception of preparation (c) (Figure 2, *B*; *r* = 0.999, *P* = 0.001). Preparations (*a*) and (*c*) appeared identical in terms of acid dissolution, cellular uptake and cellular ferritin formation *in vitro* (Figure 1), but iron from preparation (*a*) was absorbed *in vivo* at ~ 80% the efficiency of Fe(II) sulfate while, for preparation (*c*) the efficiency was only ~ 15% (Figure 2, *A*). Gastric acid solubility appeared *inversely* associated with bioavailability of the nano Fe(III) materials (*r* = − 0.90) albeit not quite significant (*P* = 0.1).

Of the 5 different nano Fe(III) materials, preparation (*a*) was best absorbed/utilised *in vivo* (Figure 2, *A*). However, when similar doses of Fe(III) chloride plus tartaric and adipic acids were dosed together *in vivo,* at the same ratios used in the synthesis of preparation (*a*), they yielded only about one third of the iron absorption [*P* = 0.004 *versus* preparation (*a*)] (Figure 2, *A*).

**Figure 3:**
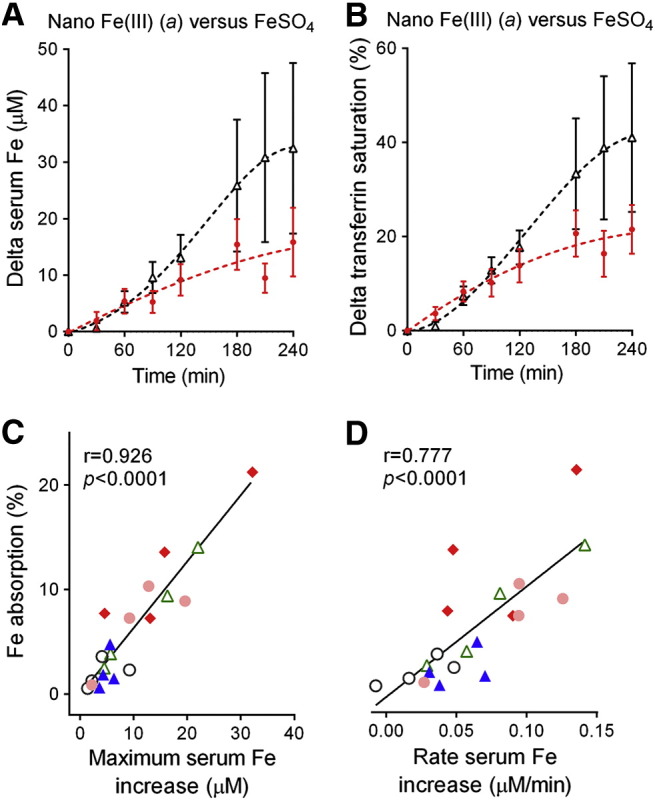
**Serum iron absorption following ingestion of a single-dose of the different Fe materials in iron-deficient women.** Serum iron increase **(A)** and transferrin saturation increase **(B)** following a single dose of nano Fe(III) preparation (*a*) (closed circles) and Fe(II) sulfate (open triangles). Values are shown as mean and error bars represent SEM (*n* = 4). Transferrin saturation was defined as serum iron divided by total iron binding capacity and expressed as a percentage. **(C-D)** Pearson's correlation between percentage of iron absorption (calculated from the red cell incorporation of ^58^Fe) and maximum serum Fe increase **(C)** or rate of serum iron increase **(D)** for the five nano Fe(III) materials. Data points correspond to each individual study participant and are colour coded to reflect the different nano Fe(III) preparations: closed diamonds, nano Fe(III) (*a*); open triangles, nano Fe(III) (*b*); open circles, nano Fe(III) (*c*); closed triangles, nano Fe(III) (*d*); closed circles, nano Fe(III) (*e*). Nano Fe(III) (*a*)*—*ligands are tartaric (T) and adipic (A) acids at a ratio 1:1:2 (T:A:Fe) and the material was dried prior to re-suspension; nano Fe(III) (*b*)—ligands are tartaric (T) and succinic (S) acids at a ratio 1:1:2 (T:S:Fe) and the material was dried prior to re-suspension; nano Fe(III) (*c*)—ligands are tartaric (T) and succinic (S) acids at a ratio 1:6:2 (T:S:Fe) and the material was dried prior to re-suspension; nano Fe(III) (*d*)—ligands are gluconic (G) and adipic (A) acids at a ratio 1:1:2 (G:A:Fe) and the material was dried prior to re-suspension; nano Fe(III) (*e*)— ligands are tartaric (T) and adipic (A) acids at a ratio 1:1:2 (T:A:Fe) and the material was used as a colloidal suspension (i.e. as synthesised) without drying (more details in Table 1).

Ferrous sulfate was not labelled so formal comparisons between this and the nano Fe(III) preparations, in terms of rate of Fe absorption *versus* absolute Fe absorption, were not possible. However, in all cases, the orally dosed nano Fe(III) materials raised serum iron levels and transferrin saturation slowly and less effectively (at 4 hours) than the same dose of iron from Fe(II) sulfate [e.g. Figure 3, *A* and *B* for nano Fe(III)]. Nonetheless, as stated above, whether these were disproportionally low when considering the absolute absorption compared to the same measures for ferrous sulfate could not be addressed. Indeed, absolute absorption of all nano Fe(III) materials (determined from the ^58^Fe red-cell incorporation) correlated with maximum serum iron increase (Figure 3, *C*; *r* = 0.926, *P* < 0.0001) and the rate of serum iron increase (Figure 3, *D*; *r* = 0.805, *P* < 0.0001), although variation for some of the nano iron formulations [for example nano iron (*a*)] was high. As such, the linear association between absolute absorption and percentage of recovery, as defined by Conway et al,^39^ is 2.2 ± 0.1 x % recovery at maximum serum Fe (*r* = 0.908, *P* < 0.0001) for nano Fe(III) whereas the published data for ferrous sulfate suggest a markedly higher slope for the same relationship (namely 8.8 ± 0.9, *r* = 0.78, *P* < 0.0001).^39^

## *In vitro* and *in vivo* toxicity assessment

Since preparation nano Fe(III) (*a*), namely iron hydroxide adipate tartrate or IHAT, appeared most promising for translation to clinical/nutritional practice, we next considered the effects of this preparation *in vitro* on cell viability and *in vivo* on colonic bacterial diversity.

**Figure 4:**
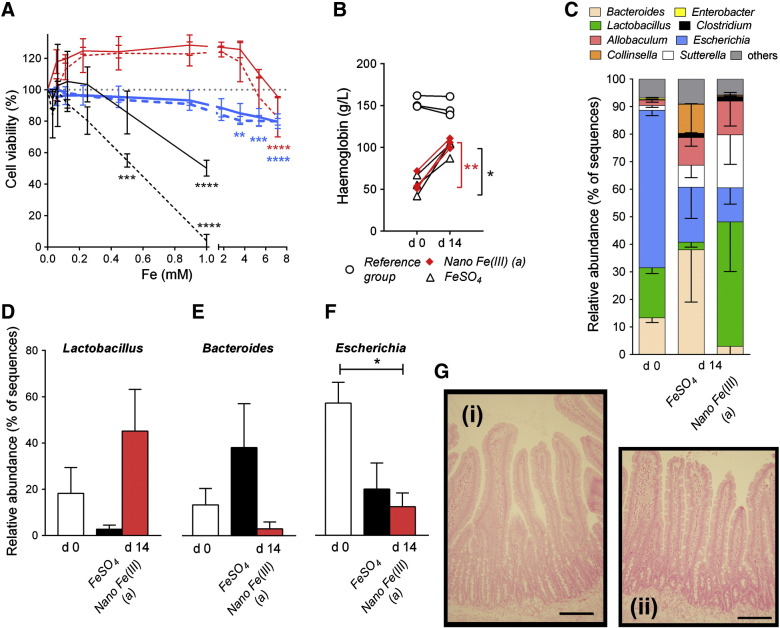
**Effects of nano Fe(III) on cell viability and the intestinal microbiome of rats. (A)** Viability of Caco-2 (red lines) and HT-29 (bold blue lines) cells exposed to increasing concentrations of Fe as nano Fe(III) (*a*) (ligands are tartaric (T) and adipic (A) acids at a ratio 1:1:2 (T:A:Fe) and the material was dried prior to re-suspension) for 24 (solid line) or 48 (dashed line) hours. Fe(II)-ascorbate (molar ratio 1:10) data in Caco-2 cells are shown in black. Results shown are mean ± SD of three independent experiments (each condition tested in triplicate wells per experiment). **, *P* < 0.01; ***, *P* < 0.001; ****, *P* < 0.0001 in relation to control cells incubated in the absence of the iron materials (100% viability). **(B)** Haemoglobin levels of anaemic Sprague–Dawley male rats following 14 days dietary supplementation with nano Fe(III) (*a*) or Fe(II) sulfate (FeSO_4_). Data are shown for each animal at baseline (d0) and after 14 days (d14) iron supplementation. Data for the reference iron-replete group (i.e. rats fed the standard iron-sufficient diet throughout) are also shown. *, *P* = 0.04; **, *P* = 0.01 corresponding to the paired *t* test between day 0 and day 14 for FeSO_4_ and nano Fe(III) (*a*), respectively. **(C)** Characterisation of the faecal microbiota at the genus level of rats receiving either Fe(II) sulfate or nano Fe(III) (*a*) at baseline (d0) and after 14 days supplementation (d14). Proportions (mean ± SEM) of the three predominant genera *Lactobacillus***(D)**, *Bacteroides***(E)** and *Escherichia***(F)**) are shown at baseline and at day 14. *, *P* = 0.03. The differences between Fe(II) sulfate and nano Fe(III) (*a*) did not reach significance for *Lactobacillus* (*P* = 0.1) or *Bacteroides* (*P* = 0.2). **(G)** Paraffin-embedded sections of the small intestine of animals supplemented with **(i)** nano Fe(III) (*a*) or **(ii)** Fe(II) sulfate without detectable iron staining (Perls' Prussian Blue). Scale bar represents 100 μm.

At therapeutically useful levels, nano Fe(III) (*a*) was not cytotoxic to either Caco-2 or HT-29 cells based on cell viability assays (Figure 4, *A*). Loss of viability was observed only for [Fe] ≥ 4 mM, which is much higher than the anticipated iron concentrations in the small intestine following supplementation (*ca.* 0.2 mM) assuming therapeutic doses of 60 mg iron up to three times a day.^40^ In contrast, Fe(II) sulfate:ascorbate (molar ratio 1:10) was found to reduce Caco-2 cell viability at Fe concentrations ≥ 0.5 mM (Figure 4, *A*).

To further investigate the effects of nano Fe(III) on the intestinal mucosa and faecal microbiota, we carried out a pilot study in rats. For 14 days following a 6 week iron depletion period, rats were fed either preparation nano Fe(III) (*a*) or Fe(II) sulfate. The preparations provided equal repletion in haemoglobin (Figure 4, *B*) and, during this period, food intake and changes in body weight were consistent with those of a non-iron deficient reference group (Supplementary Table S3).

We also compared the faecal microbiota profile of rats supplemented with preparation nano Fe(III) (*a*) to those given Fe(II) sulfate by pyrosequencing of the gene encoding 16*S* RNA. We obtained an average of 5144 sequences per animal after quality control. There were no statistically significant differences in total bacterial diversity, as assessed by Shannon's diversity and evenness indices, or in the number of total operational taxonomic units (OTUs) between the two treatment groups (Supplementary Table S4). However, the nano Fe(III) supplemented group showed an apparent increase in the proportion of *Lactobacillus* spp. and a decrease in *Bacteroides* spp. in relation to the animals supplemented with Fe(II) sulfate (Figure 4, *C*, *D*, and *E*). There was a high prevalence of the genus *Escherichia* in the iron deficient animals (day 0) and this appeared to decrease following iron supplementation with nano Fe(III) (*a*) and to some extent also with Fe(II) sulfate (Figure 4, *F*). Finally, similar to rats fed a control diet, there was no detectable iron deposition in the mucosa of the small intestine of nano Fe(III)-fed rats (Figure 4, *G*; online Supplement Figure 1).

## Discussion

Fe(III) nanoparticles deserve careful attention as potential therapeutic agents for three reasons. First, iron oxo-hydroxides represent one of the luminally-formed digestion products of dietary non-haem iron.^25^, ^41^ Secondly, dietary ferritin is a commonly ingested Fe(III) nanoparticle of protein-encapsulated ferrihydrite^31^, ^42^ and its potential role in biofortification, such as through the Global HarvestPlus initiative,^43^, ^44^, ^45^, ^46^ has gained much attention. Thirdly, nano Fe(III)-based supplements/fortificants could provide bioavailable iron and our preliminary data suggest that gastrointestinal and systemic adverse-effects may be minimised.

By doping the synthetic Fe(III) poly oxo-hydroxide structure with low molecular weight dietary ligands, we have been able to achieve a small series (a–e) of fine nanodisperse Fe(III) structures (< 10 nm diameter when aquated) with markedly differing *in vitro* solubility. These nanodisperse Fe(III) structures are composed of synthetic 2-line ferrihydrite primary particles modified and destabilised by the introduction of the organic acids, as extensively characterised elsewhere.^23^ Destabilisation of ferrihydrite primary particles in this fashion resembles that reported for the iron oxo-hydroxide core in ferritin.^31^, ^47^

Acid solubility of iron compounds has been consistently reported to be a good proxy for cellular iron uptake and *in vivo* bioabailability.^48^, ^49^, ^50^, ^51^, ^52^, ^53^, ^54^ However, for the five nano Fe(III) materials presented herein, acid solubility was *inversely* associated to their *in vitro* cellular uptake (Figure 1, *D*), which correlated with bioavailability in humans (Figure 2, *B*). To be able to draw this conclusion, it was necessary to investigate cellular uptake/utilisation of non-agglomerated and non-solubilised whole nanoparticles (i.e. assuming that they ‘survive’ gastric digestion). Hence we have used a Caco-2 cell assay optimised for our work with nano iron^25^ rather than the more sophisticated model developed by Glahn and colleagues, for example, for iron materials where solubilisation in the stomach dictates absorption.^55^

Moreover, for four of the five materials, bioavailability was clearly positively associated with cellular uptake, suggesting that direct cellular uptake/adhesion, rather than gastric dissolution, were drivers of bioavailability. Preparation (*c*), synthesised in the presence of tartaric acid and high dose succinic acid, differed (Figure 2, *B*), and we speculate that it may agglomerate to a poorly absorbable form in the gastrointestinal lumen. However, overall and consistent with what we have reported previously in rodent studies,^23^ we here demonstrate in human subjects that (i) a ligand-doped Fe(III) oxo-hydroxide nanoparticle [nano Fe(III) (*a*): that we now term ‘IHAT’ as it is based upon iron hydroxide adipate tartrate] can be absorbed ~ 80% as efficiently as the ‘gold standard’ Fe(II) sulfate and (ii) the nanostructured IHAT is ~ 3-fold better absorbed than the simple Fe(III) chloride salt even when augmented with the same ligands as IHAT (Figure 2, *A*). Furthermore, our data suggest that nanosizing and/or destabilising of the oxo-hydroxide structure is necessary for efficient bioavailability (Figure 1) and that the determinant for bioavailability is epithelial cellular adhesion/uptake of the intact nanoparticle rather than gastric acid solubility (Figure 2, *B*). This is consistent with our cellular and murine data but differs from reports where *in vitro* acid solubility (pH 1.0) predicts *in vivo* bioavailability of nanostructured Fe(III) pyrophosphates and Fe(III) oxides.^52^ However, unless molecularly destructured Fe phosphates and Fe oxides are, likely, too stable for lysosomal dissolution, even when nanosized, and thus would require gastric conditioning. The finding that a destructured nanodisperse Fe(III) oxo-hydroxide is utilised in humans with an efficiency almost equal to pure Fe(II) ions has important implications for our understanding of dietary iron digestion and absorption. Based upon murine and cellular models, we have reported that the uptake of this form of iron is via endocytosis, without prior requirement for mucosal reduction of Fe(III) to Fe(II).^23^, ^25^ Following lysosomal dissolution the iron derived from nano Fe(III) joins the common enterocyte iron pool and is exported to the systemic circulation via ferroportin according to body iron needs.^24^ This mechanism of absorption resembles that proposed for dietary ferritin and could even represent a dominant mechanism for absorption of dietary non-haem iron.^27^, ^30^, ^56^ Further work should seek to address whether it is this route or DMT-1 that enables apical uptake of most dietary non-haem iron in humans.

Nonetheless, regardless of its bioavailability and relationship to the diet, if nanodispersed Fe(III) is to be used for fortificant and supplemental purposes, its safety must be comparable or superior to current iron preparations. To this end we considered here some initial markers of potential toxicity.

One potential concern with current oral iron supplements is the generation of non-transferrin bound iron (NTBI) in the systemic circulation following oral Fe(II) sulfate^18^, ^57^, ^58^, ^59^ although the validity and interpretation of these findings remain controversial.^60^ Nonetheless, consistent with the idea that the nano Fe(III) materials have a different mechanism of absorption to soluble Fe(II) ions the appearance of iron in serum from these materials was less efficient compared to iron from Fe(II) sulfate, with lower peak iron and transferrin saturation levels (Figure 3). Whether this translates to lower NTBI or lower systemic infection risk needs to be examined, especially when matched for *absolute* iron absorption against soluble supplements.

Secondly, in cellular studies with the best-absorbed nano Fe(III) material [preparation (*a*): IHAT], we showed that unlike for Fe(II)maintained soluble with ascorbic acid,^40^ adverse cellular effects were detectable only at levels far exceeding luminal iron levels following supplementation (Figure 4, *A*). Indeed based upon a ‘worst case scenario’ of 1 mM luminal Fe concentration following oral supplementation,^40^, ^61^, ^62^ IHAT would be at a 7-fold lower concentration than is required for cellular toxicity to start. On the other hand soluble ferrous iron is clearly toxic at this 1 mM concentration.

Thirdly, deposition of ingested nanoparticles in the intestinal mucosa is a potential concern.^63^, ^64^ In the rat study presented here, there were no ultra-structural changes in the small intestine tissue or abnormal iron deposition in the intestinal mucosa for the nano Fe(III) material following 14 days of feeding (Figure 4, *G*). This is similar to the findings of Hilty et al for other nanostructured iron materials^52^ but contrary to what has been observed for some soluble Fe(III) chelates^10^, ^11^ which, again, suggests that nano iron materials may be safer to the gut than soluble iron.

Fourthly, in recent years, undesirable alterations to the intestinal microbiota have been linked to iron supplementation.^7^, ^16^, ^17^ The only human data available suggest that beneficial *Bifidobacterium* and *Lactobacillus* numbers in faeces decrease in favour of *Enterobacter* following 6-months of iron supplementation with electrolytic iron-fortified biscuits.^7^ In the small *in vivo* study presented here, faecal *Lactobacillus* numbers, far from being suppressed by the nano iron, appeared to increase, albeit not significantly with the low study numbers available (Figure 4, *D*). Indeed, these observations were from a pilot study in anaemic rats and, although consistent with the encouraging safety profile of nano iron in the cellular studies, would benefit from more detailed follow up. For example, we noted that all iron deficient animals suffered significant diarrhoea, possibly related to the high prevalence of colonic *Escherichia fergusonii*^65^ that was found in these severely iron-deplete animals (Figure 3, *F*), so an ‘otherwise healthy’ iron deficient phenotype would be of value in future work. It would also be of great interest to test the effect of IHAT/nano Fe(III) on exacerbation of colon cancer, as this is an especially worrisome artificial trait of Fe(III) chelates^10^, ^11^ and, perhaps, is averted for dietary-like nanoform iron.

In conclusion, fine structures of nano Fe(III) may be directly absorbed by the human gastrointestinal tract and are efficiently utilised as a dietary iron source. Initial observations imply superior safety of this nano Fe *versus* soluble forms of iron. Further work should carefully address safety since iron deficiency anaemia is widely prevalent and there are significant safety concerns with current forms of soluble supplemental oral iron.

## Ethical approval statement

The human study was approved by the U.K. National Research Ethics Service (06/Q0102/47). All participants signed an informed consent prior to enrolment in the study. The animal study was approved by the QIMR Berghofer Animal Ethics Committee.

## Author contributions

DIAP, SFAB, NJRF, GJA, CDV and JJP designed the research; DIAP, MFA, LKP, MAT and DMF conducted the research; DIAP and MAT analysed data; NJRF and SFAB provided the iron materials; DIAP and JJP had primary responsibility for final content. All authors read, provided input to, and approved the final manuscript.

## Appendix A Supplementary data

Supplementary Tables

Supplementary Fig. S1 **Paraffin-embedded section of the small intestine of control animals supplemented with an Fe-sufficient diet.** Image shows no detectable iron staining (Perls' Prussian Blue). Scale bar represents 100 μm.
