# Supplementary material for: Nanoparticulate iron(III) oxo-hydroxide delivers safe iron that is well absorbed and utilised in humans
Source: Nanomedicine. 2014 Nov;10(8):1877–86. doi: 10.1016/j.nano.2014.06.012 (PMC4228177; doi:10.1016/j.nano.2014.06.012)
Supplement: Supplementary file 1 — Supplementary Methods [file mmc1.pdf]

Title: Nanoparticulate iron(III) oxo-hydroxide delivers safe iron that is well absorbed and utilised in humans.

Dora I.A. Pereira, Sylvaine F.A. Bruggraber, Nuno Faria, Mani A. Tagmount, Mohamad F Aslam, David M. Frazer, Chris D. Vulpe, Gregory J. Anderson, and Jonathan J. Powell.

Supplementary Methods

Synthesis and characterisation of iron materials. The nanoparticulate ligand-modified Fe(III) poly oxo-hydroxides, here referred to as nano Fe(III), were produced using food grade reagents following the protocol described by Powell *et al*. (1). Briefly, an acidic concentrated stock solution of Fe(III) chloride was added to a solution containing GRAS (generally recognised as safe) organic acids or, in the case of unmodified Fe(III) oxo-hydroxide, to 0.9 %(w/v) potassium chloride. The initial pH of the mixture was always below 2.0, and the iron was fully solubilized as determined by ultrafiltration (M_r cut-off 3,000 Da; 10,000 x *g*, 10 min). The pH was then slowly increased by drop-wise addition of a concentrated solution of NaOH with constant agitation until the desired final pH (ca. 7.4 for nano Fe(III) and 7.4-8.2 for unmodified Fe(III) oxo-hydroxide) was attained. In the case of the nano Fe(III), the entire mixture was then oven-dried at 45° C for a minimum of 24 hours. For the insoluble unmodified Fe(III) oxo-hydroxide, the mixture was first centrifuged (6000 x *g*, 15 min), the supernatant discarded, and the solid phase dried at 45°C for a minimum of 8 hours. Fe(III) maltolate (Fe(III) maltol) was used as a soluble Fe control in the cellular iron uptake assays as previously reported (2). It was produced by mixing a solution of Fe(III) chloride with a maltol (3-hydroxy-2-methyl-4H-pyran-4-one) solution to achieve a molar ratio of Fe:maltol of 1:5. The pH of this mixture was adjusted to 7.4 with 5M NaOH.

Iron Content. The total Fe content was determined by inductively-coupled plasma optical emission spectrometry (ICP-OES JY 2000, Horiba Jobin Yvon Ltd., Stanmore, UK). Unless otherwise stated, ICP-OES Fe standards and samples were diluted in 1.1M HNO_3 to concentrations in the range 0-1000 mg_{Fe}/kg. Throughout only polystyrene and polypropylene materials were used that contained trace amounts of contaminant iron (3).

Particle size and iron phase distribution. The hydrodynamic particle size of the nano Fe(III) materials was determined by Dynamic Light Scattering (DLS) using a Zetasizer, Nanoseries Nano ZS (Malvern Instruments Ltd., Worcestershire, UK). Any large agglomerates (always

less than 10% of total iron) were removed by centrifugation (10,000 *xg*, 5 min) and DLS was used to measure the size of particles in suspension immediately following centrifugation in the medium used for the cellular assays. DLS data are reported as average particle diameter, dv0.9 (diameter under which 90 percent of the population falls), and dv0.1 (diameter under which 10 percent of the population falls).

For each of the nano Fe(III) materials suspended in the medium used for the Caco-2 assays, fractionation of the Fe into percentages of nanoparticulate, microparticulate and soluble Fe was achieved by centrifugation and ultrafiltration. Preparations were centrifuged (10,000 *xg*, 5 min) and the sediment considered as the microparticulate fraction. In order to isolate the soluble Fe and to distinguish it from nanoparticulate Fe, the supernatant was further ultrafiltered (M_r cut-off 3,000 Da; 10,000 x *g*, 10 min). The Fe content of total, supernatant and ultrafiltrate fractions was determined by ICP-OES as described above, and microparticulate, nanoparticulate and soluble iron were expressed as percentage ± SD in relation to total Fe content as follows:

[(%) Fe microparticulate] = [(Total Fe – Fe $_{supernatant}$)/Total Fe] x 100

[(%) Fe nanoparticulate] = [(Fe $_{supernatant}$ – Fe $_{ultrafiltrate}$)/Total Fe] x 100

[(%) Fe soluble] = [(Fe $_{ultrafiltrate}$)/Total Fe] x 100

Acid lability assay. The iron materials were suspended in 0.15 M NaCl to an iron concentration of 2 mM and incubated for 95 min at room temperature. The pH was automatically maintained at pH 3.0 by an autotitrator (Metrohm, Malvern Instruments, UK) with 1 M NaOH and 1 M HCl. Samples were collected at 5, 10, 15, 35, 55 and 95 min for the assessment of solubility. The assay was performed in duplicate for each material. The soluble Fe fraction was determined as detailed above.

Caco-2 iron uptake studies. Caco-2 cells (passages 24 – 45) were grown at 37°C in an atmosphere of 5% CO_2 and 95% air at a relative humidity of approximately 95%. Cells were maintained in T-75 flasks using Minimum Essential Medium (MEM, PAA Laboratories, Yeovil, UK) supplemented with 10% foetal bovine serum (FBS "Gold", PAA Laboratories), 1% penicillin/streptomycin and 1% fungizone (Invitrogen, Paisley, UK). The growth medium was changed every 2-3 days. Cells were passaged at 70- 80% confluence using trypsin 0.25%(w/v) in 1 mM ethylenediaminetetraacetic acid (EDTA) solution.

The medium for the Caco-2 iron uptake experiments consisted of a balanced salt solution (BSS) containing 130 mM NaCl, 10 mM KCl, 1 mM $MgSO_4$, 5 mM glucose and 1 mM

$CaCl_2$ in 10 mM PIPES buffer, pH 7.4. This medium was used to avoid agglomeration of the novel nanoparticulate iron materials, and this was assessed by the fractionation protocol described above. Cells were seeded at approximately 1.8 x 10^5 cells/mL onto 6-well cell culture plates. The Caco-2 cells were maintained under the culture conditions described above and used for uptake experiments at 11 or 12 days post-seeding (under these culture conditions the cell monolayer is fully confluent at day 5 post-seeding and differentiated at day 10 post-seeding (4)). Approximately 16 hours prior to the experiments, the growth medium was replaced by non-supplemented MEM, i.e. without FBS or antibiotics, and cells were returned to the incubator. Monolayer integrity was assessed by trans-epithelial electrical resistance (TEER) measurements prior to each experiment and resistance was maintained following the serum free incubation period (data not shown). On the day of the experiment the Caco-2 cell monolayer was washed once with pre-warmed Dulbecco's Phosphate-Buffered Saline (DPBS) and incubated with BSS supplemented with the different iron materials ([Fe] = 0.5 mM) or control uptake medium (non-supplemented BSS) for 1 hour at 37°C. For each experiment, every condition was investigated in triplicate wells, and each experiment was repeated 3 times. Following the iron incubation period, the uptake medium was removed, the cell monolayer was washed three times with PBS-EDTA (2 mM) to remove any iron loosely adherent to the cell membrane, and fresh non-supplemented MEM was added. The cells were returned to the incubator for an additional 23 hours to allow for ferritin formation, as optimised previously (2, 5). At the end of the incubation period, the cells were washed and lysed with Mammalian Protein Extraction Reagent (MPER®, Thermo Fisher Scientific, Cramlington, UK). Cell debris was removed by centrifugation (5 min, 16,000 x *g*) and the supernatant was used for ferritin, cellular iron and cellular protein analysis.

Analysis. Cell lysate supernatants were analysed for cellular Fe content by ICP-OES as described above, using standards in the range 0-1000 μg_{Fe}/Kg, and for ferritin content using the commercial enzyme-linked immunosorbent assay (ELISA) kit “Spectro Ferritin” (ATI Atlas, Chichester, UK). Total cell protein content was determined with the Non-Interfering Protein Assay™ (NIPA™, Calbiochem/Merck, Nottingham, UK).

Cell viability assay. Caco-2 cells and HT-29 cells were grown at 37°C, in an atmosphere of 5% CO_2 and 95% air at a relative humidity of approximately 95%, in DMEM/F12 medium (Invitrogen, Grand Island, Nebraska, USA) supplemented with antibiotics (1%

penicillin/streptomycin) and 20% FBS (Invitrogen). Cells were seeded at 1.5 x 10^4 cells/mL in 96-well plates (100 µL/well) and incubated for 24 hours prior to exposure to varying concentrations of the different iron materials ([Fe] = 0.05-7 mM). Immediately prior to the assay, the Fe materials were re-suspended at the required concentration in complete cell growth medium and the cells were incubated at 37°C with this supplemented medium for 24 or 48 hours. Cell viability was evaluated using the CellTiter 96® AQueous One Solution Cell Proliferation Assay (MTS) (Promega, Madison, Wisconsin, USA). Briefly, following the incubation period, the medium in each well was replaced with 120 µL of a mixture of 5 volumes medium to 1 volume MTS solution and the cells were incubated for a further 60 to 90 min. The plates were centrifuged for 10 min at ca. 600 x *g*. The supernatant (100 µL) was transferred to a new plate and the absorbance was read at 490 nm. For each of the three independent experiments, the absorbances of three replicate wells were averaged and the survival percentage was determined using the absorbance of the negative control (cells incubated in the absence of the iron material) as 100 % survival. Fe(II)-ascorbate (1:10) was not tested at [Fe] > 1mM as ascorbate interferes in the MTS assay at concentrations above 10mM.

Animal study. Animals had unlimited access to deionised water throughout. Food intake and body weights were measured three times a week during the study. Faecal samples were collected from each animal at the start (Day 0) and end (Day 14) of the test diet administration period. After the study termination (day 15) the rats were anaesthetized with a single intraperitoneal injection of xylazine (8 mg/kg) and ketamine (44 mg/kg) and the animals were euthanized by exsanguination. Enhanced Perls' Prussian Blue stain was performed on formalin fixed paraffin embedded sections of the duodenum as described previously (6).

Faecal microbiota analysis using 454 pyrosequencing. DNA was extracted using a QIAamp DNA Stool Mini Kit (Qiagen, VIC, Australia) as per the manufacturer's protocol. Gut microbiota composition was analysed by 454 pyrosequencing of the total faecal community 16S rRNA gene. This analysis was performed by Molecular Research LP (www.mrdnalab.com, Shallowater, Texas, USA). The sequence data were processed using the Molecular Research LP proprietary analysis pipeline using a quality threshold of Q25. Sequences were depleted of barcodes and primers and short sequences < 200bp. Sequences with ambiguous base calls and sequences with homopolymer runs exceeding 6bp were also

removed. Sequences were then ‘denoised’ and chimeras removed. Operational taxonomic units (OTU) were defined after removal of singleton sequences (sequences appearing only once in the whole dataset) with a cluster threshold set at 3% divergence (i.e. 97% similarity) to minimize the influence of sequencing errors (7-13). OTU were then taxonomically classified using BLASTn against a curated GreenGenes database (14) and compiled at each taxonomic level.

Human study

Design and Subjects. Potential participants were recruited from the local community and screened at the MRC Human Nutrition Research (MRC HNR) facility for determination of iron status (haemoglobin, serum ferritin, transferrin saturation), indicators of acute inflammation (C-reactive protein) and biochemical markers of liver function (albumin, ALT, γGT, total bilirubin, ALP) and kidney function (urea, creatinine). The eligibility criteria were iron deficiency (defined as serum ferritin less than 12 μg/L) or mild-moderate iron deficiency anaemia (defined as haemoglobin between 10-11.9 g/dL plus either a serum ferritin less than 20 μg/L or transferrin saturation < 10%) (15). We excluded subjects with severe anaemia, or other disorders that may affect iron absorption and metabolism (e.g. haemochromatosis or self-reported coeliac disease), as well as adhering to standard exclusion criteria at the Institute (e.g. self-reported pregnancy, lactation, recent surgery, chronic disease). Subjects with serum CRP values of 10 mg/L or above were excluded (16).

Eligible participants were invited to two study visits (day 1 and day 14). At each visit, a blood sample was taken to measure iron-related parameters, including serum ferritin, serum iron and transferrin saturation, CRP, full blood count, and erythrocyte incorporation of ^{58}Fe as explained below. On day 1 of the study, the participants were given one methyl cellulose capsule containing one of the test iron formulations to be taken with a light breakfast consisting of water and two slices of white bread with jam. Serial serum iron levels were obtained at 30, 60, 90, 120, 180, 210 and 240 minutes after ingestion of the iron capsule. Fourteen days later the participants returned for the second study visit where they ingested one Fe(II) sulfate tablet. Sampling was then carried out as described above. The test Fe(III) materials were labelled with stable isotopic ^{58}Fe, as described below, and each volunteer acted as her own control by also taking the Fe(II) sulfate on the second occasion. Absorption of the nano Fe(III) materials was determined by erythrocyte incorporation of ^{58}Fe (17) as described below. Fe(II) sulfate was not labelled with ^{58}Fe and iron absorption, in this case,

was calculated from short-term changes in serum iron levels using two validated algorithms (18, 19).

Laboratory analysis. Haemoglobin levels, as well as liver and kidney function parameters were measured using standard clinical methodology at Cambridge University Hospitals NHS Foundation Trust. Serum ferritin was measured using a latex-enhanced immunonephelometric method on the BN ProSpec® system (Siemens Healthcare, Surrey, U.K.). C-reactive protein (conventional) was measured using a particle-enhanced turbidimetric immunoassay on the Dimension® Xpand chemistry analyser (Siemens Healthcare, Surrey, U.K.). The normal reference range for the conventional CRP assay is below 10 mg/L (16) . Soluble transferrin receptor was measured in serum by a sandwich ELISA from Ramco Laboratories (ATI Atlas, Chichester, UK) following the manufacturer's protocol.

Isotope ratio measurement and calculation of iron absorption. The stable iron isotope ^{58}Fe (>93%) was purchased in elemental form (Chemgas, Boulogne-Billancourt, France). Iron isotope solutions were prepared as Fe(III) chloride by dissolving the metal in $HCl:H_2O_2$ (8:1 ratio) at 60°C. These iron isotope solutions were then added, to reach a final amount of approximately 2 mg ^{58}Fe per 60 mg iron dose, to the Fe(III) chloride acidified solution used for the synthesis of the different iron materials as detailed above (i.e. providing a ^{58}Fe label of 2 mg per dose). Iron isotope ratios were measured in whole blood at baseline (day 0) and 14 days after dosing by inductively-coupled plasma mass spectrometry (ICP-MS) as previously described. (17). Briefly, whole blood was diluted (100-fold) in a mixture of 0.5 %(v/v) Triton X-100, 1%(v/v) butan-1-ol, 0.5%(v/v) ammonia, and 0.008%(v/v) nitric acid. The iron isotope ratios were measured with an Elan DRCplus ICP-MS (Perkin Elmer Sciex, Beaconsfield, UK). The results were expressed as the ratio of $^{58}Fe:^{57}Fe$. Sample pairs for each subject (day 0 vs. day 14) were analysed consecutively. Iron absorption from the nano Fe(III) was calculated from the red cell incorporation of the ^{58}Fe isotope 14 days after the oral dose as described previously (17, 20, 21). It was assumed that 80% of absorbed iron was incorporated into red blood cells (22) and that for women the blood volume is 69.6 mL/kg (23-25).

As stated above, for Fe(II) sulfate, iron absorption was calculated from short-term changes in serum iron levels using two validated algorithms (18, 19). The serum iron increase at 180 minutes following the single-dose of ferrous sulfate was used in these calculations and the mean value from the two algorithms was taken.

Relative bioavailability values (RBV) for nano Fe(III) were determined by dividing iron absorption from nano Fe(III) by iron absorption from ferrous sulfate for each study subject. RBV were expressed in percentage.

Statistical analysis. All statistical analyses were performed using GraphPad Prism 6 for Windows (GraphPad Software, San Diego, California, USA). Unless otherwise stated, results are presented as means with standard deviations (SD). Unpaired t-tests were used to compare means for relative bioavailability values to Fe(II) sulfate (%RBV) in the human study. Paired t-tests were used to compare haemoglobin for each iron material at the 2 time-points in the animal study. Analysis of variance (one-way ANOVA) with the Sidak's multiple comparisons test was used to compare cellular iron, cellular ferritin, bacteria genera and haemoglobin levels between the two different iron materials (cell studies) or the diets supplemented with the two different iron materials (animal study). Repeated measures ANOVA (two-way ANOVA) with Bonferroni post-test was used to compare means for cell viability at different iron concentrations and for serum iron increase at different timepoints. The rate of serum iron increase was taken as the slope of the tangential line through the initial time-points of the serum iron curve (i.e. for the initial linear portion of the curve; usually up to 120 min). Analysis of covariance (ANCOVA) was used to statistically compare the rates of serum iron increase between nano Fe(III) and Fe(II) sulfate. Pearson correlation was used to assess linear associations. Significance was considered to be reached at $p<0.05$.

Supplementary References

1. Powell JJ, Bruggraber SF, Faria N, Poots LK, Hondow N, Pennycook TJ, et al. A nano-disperse ferritin-core mimetic that efficiently corrects anaemia without luminal iron redox activity. Nanomedicine: Nanotechnology, Biology and Medicine. 2014 Jan 3.
2. Mergler BI, Roth E, Bruggraber SFA, Powell JJ, Pereira DIA. Development of the Caco-2 Model for Assessment of Iron Absorption and Utilisation at Supplemental Levels. Journal of Pharmacy and Nutrition Sciences. 2012;2(1):27-34.
3. Reimann C, Siewers U, Skarphagen H, Banks D. Does bottle type and acid-washing influence trace element analyses by ICP-MS on water samples? A test covering 62 elements and four bottle types: high density polyethene (HDPE), polypropene (PP), fluorinated ethene propene copolymer(FEP) and perfluoroalkoxy polymer(PFA). Science of the Total Environment. 1999 Oct 1;239(1-3):111-30.
4. Pereira DI, Mergler BI, Faria N, Bruggraber SF, Aslam MF, Poots LK, et al. Caco-2 Cell Acquisition of Dietary Iron(III) Invokes a Nanoparticulate Endocytic Pathway. PLoS One. 2013;8(11):e81250.
5. Mergler BI. Enterocyte uptake of nanoparticulate iron(III) oxo-hydroxide [PhD Thesis]. U.K.: University of Cambridge; 2012.

6. Frazer DM, Inglis HR, Wilkins SJ, Millard KN, Steele TM, McLaren GD, et al. Delayed hepcidin response explains the lag period in iron absorption following a stimulus to increase erythropoiesis. Gut. 2004 Oct;53(10):1509-15.
7. Edgar RC. Search and clustering orders of magnitude faster than BLAST. Bioinformatics. 2010 Oct 1;26(19):2460-1.
8. Dowd SE, Callaway TR, Wolcott RD, Sun Y, McKeehan T, Hagevoort RG, et al. Evaluation of the bacterial diversity in the feces of cattle using 16S rDNA bacterial tag-encoded FLX amplicon pyrosequencing (bTEFAP). BMC microbiology. 2008;8:125.
9. Dowd SE, Delton Hanson J, Rees E, Wolcott RD, Zischau AM, Sun Y, et al. Survey of fungi and yeast in polymicrobial infections in chronic wounds. Journal of wound care. [Case Reports]. 2011 Jan;20(1):40-7.
10. Dowd SE, Sun Y, Wolcott RD, Domingo A, Carroll JA. Bacterial tag-encoded FLX amplicon pyrosequencing (bTEFAP) for microbiome studies: bacterial diversity in the ileum of newly weaned Salmonella-infected pigs. Foodborne pathogens and disease. 2008 Aug;5(4):459-72.
11. Eren AM, Zozaya M, Taylor CM, Dowd SE, Martin DH, Ferris MJ. Exploring the diversity of Gardnerella vaginalis in the genitourinary tract microbiota of monogamous couples through subtle nucleotide variation. PloS one. [Research Support, N.I.H., Extramural Research Support, Non-U.S. Gov't]. 2011;6(10):e26732.
12. Capone KA, Dowd SE, Stamatas GN, Nikolovski J. Diversity of the human skin microbiome early in life. The Journal of investigative dermatology. [Research Support, Non-U.S. Gov't]. 2011 Oct;131(10):2026-32.
13. Swanson KS, Dowd SE, Suchodolski JS, Middelbos IS, Vester BM, Barry KA, et al. Phylogenetic and gene-centric metagenomics of the canine intestinal microbiome reveals similarities with humans and mice. The ISME journal. 2011 Apr;5(4):639-49.
14. DeSantis TZ, Hugenholtz P, Larsen N, Rojas M, Brodie EL, Keller K, et al. Greengenes, a chimera-checked 16S rRNA gene database and workbench compatible with ARB. Applied and environmental microbiology. [Research Support, U.S. Gov't, Non-P.H.S.]. 2006 Jul;72(7):5069-72.
15. WHO. Iron Deficiency Anaemia: Assessment, Prevention and Control. Geneva, Switzerland: World Health Organization2001.
16. FDA. Review Criteria for Assessment of C-Reactive Protein (CRP), High Sensitivity C-Reactive Protein (hsCRP) and Cardiac C-Reactive Protein (cCRP) Assays. Food and Drug Administration; 2005 [updated 22/09/200518/06/2014]; Available from: http://www.fda.gov/downloads/MedicalDevices/DeviceRegulationandGuidance/GuidanceDocuments/ucm071017.pdf.
17. Faria N, Winship PD, Weiss DJ, Coles BJ, Schoenberg R, Hutchison C, et al. Development of DRC-ICP-MS methodology for the rapid determination of Fe-58 erythrocyte incorporation in human iron absorption studies. Journal of Analytical Atomic Spectrometry. 2011 2011;26(8):1648-52.
18. Conway RE, Geissler CA, Hider RC, Thompson RP, Powell JJ. Serum iron curves can be used to estimate dietary iron bioavailability in humans. J Nutr. 2006 Jul;136(7):1910-4.
19. Hoppe M, Hulthen L, Hallberg L. The validation of using serum iron increase to measure iron absorption in human subjects. Br J Nutr. 2004 Sep;92(3):485-8.
20. Abrams SA. Using stable isotopes to assess mineral absorption and utilization by children. Am J Clin Nutr. 1999 Dec;70(6):955-64.
21. Kastenmayer P, Davidsson L, Galan P, Cherouvrier F, Hercberg S, Hurrell RF. A double stable isotope technique for measuring iron absorption in infants. Br J Nutr. 1994 Mar;71(3):411-24.

22. Hosain F, Marsaglia G, Finch CA. Blood ferrokinetics in normal man. J Clin Invest. 1967 Jan;46(1):1-9.
23. Wennesland R, Brown E, Hopper J, Jr., Hodges JL, Jr., Guttentag OE, Scott KG, et al. Red cell, plasma and blood volume in healthy men measured by radiochromium (Cr51) cell tagging and hematocrit: influence of age, somatotype and habits of physical activity on the variance after regression of volumes to height and weight combined. J Clin Invest. 1959 Jul;38(7):1065-77.
24. Brown E, Hopper J, Jr., Hodges JL, Jr., Bradley B, Wennesland R, Yamauchi H. Red cell, plasma, and blood volume in the healthy women measured by radiochromium cell-labeling and hematocrit. J Clin Invest. 1962 Dec;41:2182-90.
25. Lentner C, editor. Geigy Scientific Tables: Physical Chemistry, Blood, Haematology, Somatometric Data. 8th edition ed: Ciba Geigy 1984.
