# Supplementary material for: Nanoparticulate iron(III) oxo-hydroxide delivers safe iron that is well absorbed and utilised in humans
Source: Nanomedicine. 2014 Nov;10(8):1877–86. doi: 10.1016/j.nano.2014.06.012 (PMC4228177; doi:10.1016/j.nano.2014.06.012)
Supplement: Supplementary file 2 — Supplementary Tables [file mmc2.pdf]

Title: Nanoparticulate iron(III) oxo-hydroxide delivers safe iron that is well absorbed and utilised in humans.

Dora I.A. Pereira, Sylvaine F.A. Bruggraber, Nuno Faria, Mani A. Tagmount, Mohamad F Aslam, David M. Frazer, Chris D. Vulpe, Gregory J. Anderson, and Jonathan J. Powell.

Supplementary Tables

Supplementary Table S1. Composition of the iron deficient basal diet.

Ingredient	g/kg$_{diet}$	Ingredient	g/kg$_{diet}$	Ingredient	g/kg$_{diet}$
Dried skim milk	650	$CuSO_4.5H_2O$	0.0816	Folic acid	0.001
Sucrose	205	Fe [i]	----	*p*-Aminobenzoic acid	0.01
Fat (lard)	99	Ca panthothenate	0.012	Biotin	0.0002
Dextrose	30	Pyridoxine	0.005	Inositol	0.2
NaCl	6	Riboflavin	0.005	Vitamin B12	0.000015
Choline dihydrogen citrate	5	Thiamine hydrochloride	0.01	Vitamin A palmitate	0.008
$NaIO_3.H_2O$	0.660	Nicotinic acid	0.01	Calciferol	0.001
$MnSO_4.4H_2O$	0.324	Vitamin K	0.001	α-Tocopherol (35%)	1 mL

i- the control diet was supplemented with Fe(II) ammonium sulfate and the test diets with Fe(II) sulfate or Nano Fe(III) (a) in the levels presented in supplementary Table S3.

[Valberg LS, Taylor KB, Witts LJ, Richards WC. The effect of iron deficiency on the stomach of the rat. Br J Nutr. 1961;15:473-80]

Supplementary Table S2. Demographics, anthropometric and baseline iron status characteristics of enrolled subjects. Results presented as mean (min,max).

**Test Compound**	**N**	**Age (years)**	**BMI (kg/m^2)**	**CRP (mg/dL)**	**Serum Ferritin (µg/L)**	**Haemoglobin (g/dL)**	**sTfr (µg/mL)**
Nano Fe(III) (*a*)	4	24 (19,40)	22.4 (17.1, 29.7)	2.4 (0.7, 6.2)	7.7 (2.7, 12.6)	12.4 (11.3, 13)	6.9 (4.0,17.2)
Nano Fe(III) (*b*)	4	23 (21,27)	22.5 (19.6, 28.1)	2.2 (1.4, 4.4)	5.1 (3.9, 9.1)	12.1 (10.8, 12.2)	5.9 (4.1,7.6)
Nano Fe(III) (*c*)	4	25 (22,28)	20.2 (18.8, 26.2)	1.4 (0.6, 2.2)	11.1 (2.8, 57.7)	12.4 (11.5, 13.3)	4.9 (3.3,5.7)
Nano Fe(III) (*d*)	4	23 (21, 27)	22.0 (18.1, 27.2)	1.8 (1.1, 3.6)	6.9 (2.5, 20.3)	12.5 (11.5, 13.6)	7.1 (4.4,7.7)
Nano Fe(III) (*e*)	4	28 (19,30)	22.7 (19.4, 24.3)	1.1 (1.1, 1.15)	5.0 (2.4, 16.3)	11.7 (10.7, 12.5)	5.8 (3.8,7.7)
$Fe(III)(OH)_3$ [i]	2	26 (23, 29)	19.4 (18.7, 20.0)	2.9 (1.1, 4.6)	10.5 (6.6, 14.5)	11.6 (11.2, 12.0)	4.9 (4.1,5.6)
$FeCl_3$+Tart+Ad [ii]	4	28 (24, 33)	23.1 (19.9, 25.4)	1.2 (1.1, 5.8)	13.7 (12.8, 23.3)	12.0 (11.4, 12.8)	4.0 (2.7,5.4)
Fe(II) sulfate	26	25 (19, 40)	22.2 (17.1, 29.7)	2.2 (0.62, 5.5)	9.0 (2.2, 56.3)	11.9 (10.2, 13.2)	6.2 (2.8,13.9)

i- Refers to standard unmodified Fe(III) oxo-hydroxide (i.e. synthetic 2-line ferrihydrite).

ii- Refers to the unformulated mixture of the raw materials used to synthesise nano Fe(III) (*a*): Fe(III) chloride, tartaric acid and adipic acid in the molar ratio 2:1:1.

CRP, C-reactive protein; sTfr, soluble transferrin receptor.

Supplementary Table S3. Fe supplementation level, Fe intake, bodyweight gain and haemoglobin change during 14 days repletion period in rats.

Diet/ Group	N	Diet Fe content [i] (mg Fe/kg diet)	Total Fe intake (mg/14 d)	Food intake [ii] (g/ 15 d)	Baseline Hb [iii] (g/L)	Bodyweight change [ii] (g/14 d)
Fe(II) sulfate	3	17.9 ± 18.3	5.2 ± 0.9	288 ± 50	55 ± 12	80 ± 37
Nano Fe(III) (*a*)	3	23.8 ± 5.8	7.9 ± 0.2	331 ± 9	59 ± 11	88 ± 10
Reference group (Fe-sufficient)	3	103.7 ± 18.8	38 ± 4	369 ± 40	154 ± 7	87 ± 3

i- As analysed by inductively-coupled plasma optical emission spectroscopy (ICP-OES) following microwave digestion of the prepared diets (n=5 independent digests, n=3 replicates per digest)

ii- There were no significant differences in the food intake and bodyweight gain for the animals in the test and control diets.

iii- The baseline haemoglobin was not significantly different between the Fe(II) sulfate and the Nano Fe(III) (*a*) groups, but was significantly lower than the one for the reference group (Fe-sufficient) ($p<0.001$).

Suplementary Table S4. Microbial diversity indices computed on all species-based taxonomic units. Data are mean (SD).

**Diet/Group**	**N**	**Richness (S)**	**Shannon diversity index (H')**	**Shannon evenness index (E_H)**	**Number of sequences**
Day 0 (pre-treatment)	6	27 (5)	1.3 (0.43)	0.40 (0.12)	5675 (2073)
Fe(II) sulfate at day 14	3	29 (6)	1.9 (0.28)	0.56 (0.05)	2975 (317)
Nano Fe(III) (*a*) at day 14	3	27 (2)	1.5 (0.33)	0.45 (0.09)	6252 (3139)

$H' = -\sum_{i=1}^{S} pi \ln(pi)$ and $E_H = \frac{H\prime}{S}$ where S, is the total number of species per sample and pi, is the proportional abundance of each species per sample [Godden JW, Bajorath J. Shannon entropy--a novel concept in molecular descriptor and diversity analysis. J Mol Graph Model. 2000 Feb;18(1):73-6 & Hariri A, Weber B, Olmsted J, 3rd. On the validity of Shannon-information calculations for molecular biological sequences. J Theor Biol. 1990 Nov 21;147(2):235-54].
