# Supplementary material for: Nanoparticulate iron(III) oxo-hydroxide delivers safe iron that is well absorbed and utilised in humans
Source: Nanomedicine. 2014 Nov;10(8):1877–86. doi: 10.1016/j.nano.2014.06.012 (PMC4228177; doi:10.1016/j.nano.2014.06.012)

Supplementary Figure S1. Paraffin-embedded section of the small intestine of control animals supplemented with an Fe-sufficient diet. Image shows no detectable iron staining (Perls' Prussian Blue).
Scale bar represents 100 µm.

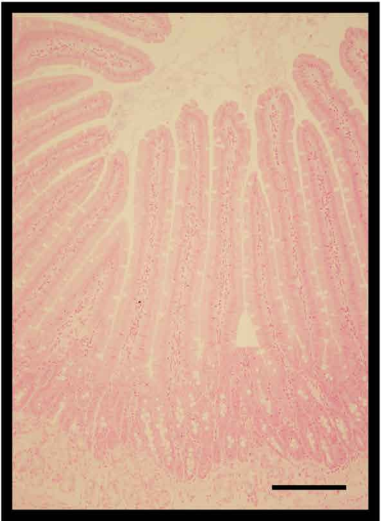

Supplement: Supplementary Fig. S1 — Paraffin-embedded section of the small intestine of control animals supplemented with an Fe-sufficient diet. Image shows no detectable iron staining (Perls' Prussian Blue). Scale bar represents 100 μm. [file mmc3.pdf]
